# Agroforestry in Madagascar: past, present, and future

**DOI:** 10.1007/s10457-024-00975-y

**Published:** 2024-05-10

**Authors:** R. Ntsiva N. Andriatsitohaina, Patrick Laby, Jorge C. Llopis, Dominic A. Martin

**Affiliations:** 1 Mention Foresterie et Environnement, Ecole Supérieure des Sciences Agronomiques, Université d’Antananarivo, Antananarivo, Madagascar; 2 School of Natural Sciences, Bangor University, Bangor, UK; 3 Centre for Development and Environment, University of Bern, Bern, Switzerland; 4 Department of Geography, University of Zurich, Zurich, Switzerland; 5 Institute of Plant Sciences, University of Bern, Bern, Switzerland

**Keywords:** Agronomy, History, Cash crops, Ecosystem services, Sustainable development, Review

## Abstract

Agroforestry systems promise a high multifunctionality providing cash and subsistence yields as well as other ecosystem services. Such land systems may be particularly promising for smallholders in tropical landscapes due to high labour intensity and productivity on limited land. Focusing on Madagascar, we here describe the history of agroforestry in the country and review the current literature on agroforestry outcomes as well as factors promoting and hindering agroforest establishment and maintenance. From this, we discuss the potential future of agroforestry in Madagascar. Historically, many crops farmed today in agroforestry systems were originally introduced as plantation crops, mostly in the nineteenth century. Since then, people co-opted these crops into mixed agroforestry systems, often focusing on clove, vanilla, coffee, or cocoa in combination with fruit trees or, for clove, with livestock. Other crops are also integrated, but shares are comparatively low. Overall, 27.4% of Malagasy exports are crops typically farmed in agroforestry systems, providing income for at least 500,000 farmers. Outcomes of agroforestry for biodiversity and ecosystem services are commonly researched, showing benefits over annual crops and monocultures. Social-economic outcomes, including yields, are more scarcely researched, but findings point towards financial benefits for smallholder farmers and a sense of community and collective memory. However, findings emphasize that research gaps remain in terms of geographic and crop coverage, also for ecological outcomes. Looking to the future, we highlight the need to overcome hurdles such as land tenure insecurity, financial barriers to implementation, and unstable value chains to scale agroforestry in Madagascar to the benefit of multifunctional land systems and human wellbeing.

**Supplementary Information:**

The online version contains supplementary material available at 10.1007/s10457-024-00975-y.

## Introduction

Agroforests, or land systems where perennial woody plants are deliberately integrated with agricultural crops or livestock (FAO 2023), support the livelihoods of millions globally, making them the economic backbone of a number of countries (Duffy et al. 2021; Lin et al. 2021). Agroforestry is also renowned for achieving multifunctionality and for sustaining and diversifying smallholder and non-smallholder production, which together can enhance social, economic and environmental benefits (Leakey 2017; Castle et al. 2021).

Socio-economically, agroforestry allows the diversification of crops, which can improve system yields and may enable harvests throughout the year, which in turn can help smoothen incidences of food and income insecurity (Mariel et al. 2021a, b). Further, agroforestry can increase household income, improving farming families’ well-being (Castle et al. 2021; Hajjar et al. 2021). For example, it has been shown that farmers with cash crop agroforestry tend to be more able to send their children to schools, or to build better houses, than their counterparts not engaged into agroforestry (Llopis et al. 2020). Besides providing valuable timber for construction and firewood (Iiyama et al. 2014), agroforestry systems also supply different tree-derived products that can be sold, such as leaves for medicines and fruits (Thevs et al. 2022). Furthermore, agroforestry supports many socio-cultural benefits, such as the aesthetic value of trees or their role as boundary markers (Gassner and Dobie 2022). Agroforestry systems can also represent the cultural identity of a community (Llopis et al. 2020; Osterhoudt 2018), and can play a central role in shaping and sustaining the identity and sense of place of communities across the globe (Smith and Mbow 2014).

From an environmental perspective, agroforestry can, for example, increase drought resistance (Nair 2011) and reduce wind and water soil erosion (Minang et al. 2014). In addition, agroforestry favors high rates of biodiversity, both above and below ground (De Beenhouwer et al. 2013). In sum, agroforestry systems can contribute to sustainable development by protecting natural resources for future generations (Smith and Mbow 2014; Castle et al. 2021).

Madagascar is one of the countries where agroforestry plays a central socio-economic, cultural, and environmental role. Madagascar is a major producer of some of the most demanded agroforestry crops, such as vanilla, clove, coffee, cocoa, and lychee, which constitute a critical input to the country's economy (FAO 2022). Agroforestry crops’ production and trade economically support hundreds of thousands of households across Madagascar, while also being a central element of local socio-cultural practices and worldviews (Osterhoudt 2018; Mariel et al. 2021a, b; Carrière et al. 2021; Llopis et al. 2021). Further, forests in Madagascar contain a biodiversity wealth nearly unparalleled in the world (Goodman and Benstead 2005), with much of these forest under severe pressures (Jones et al. 2019), to a large extent due to the expansion of subsistence agriculture (Zaehringer et al. 2017; Waeber et al. 2015). In this context, agroforestry can play a key role in forest conservation and livelihood support at the forest edge (Martin et al. 2022).

Despite the central economic and cultural significance of agroforestry systems in Madagascar and its future potential for meeting development and conservation goals in the country, so far, no synthesis has been conducted on its historical evolution, its current state, and desirable future pathways. Such a synthesis can be useful for building on existing knowledge about diverse crops introduced to promote agroforestry, to plan conservation and development interventions, including restoration activities, and to help discuss and clarify policies aiming at harnessing the potential benefits that agroforestry systems can provide for nature stewardship.

**Fig. 1:**
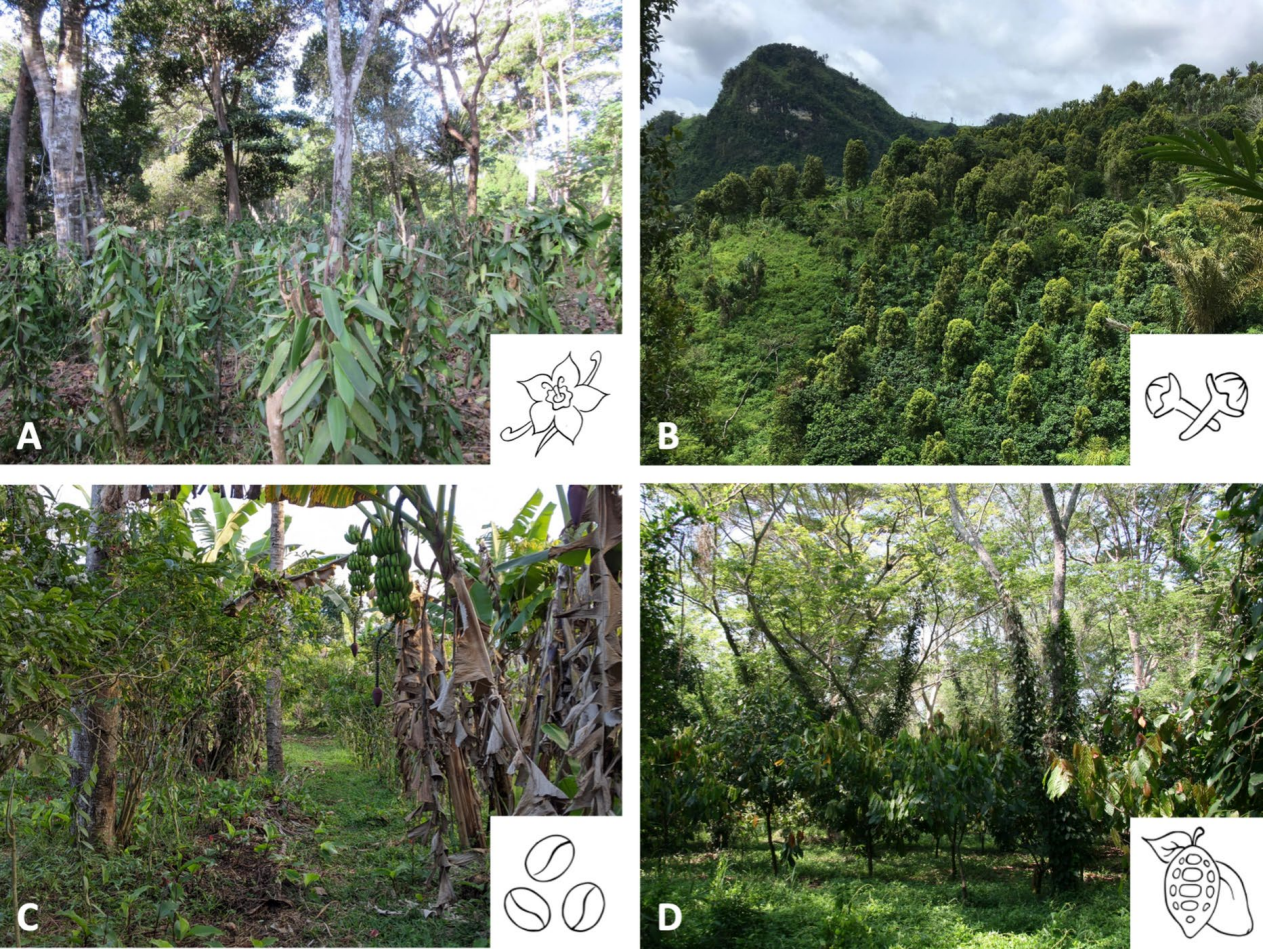
Examples of agroforestry systems in Madagascar. **A** Vanilla agroforestry system, SAVA region, northeastern Madagascar (Photo: Dirk Hölscher). **B** Clove agroforestry system, Analanjirofo region, northeastern Madagascar (Photo: R. Ntsiva N. Andriatsitohaina). **C** Coffee agroforest intercropped with banana and coconut, SAVA region, northeastern Madagascar (Photo: James Herrera). **D** Cocoa-Pepper agroforestry system, Diana region, northwestern Madagascar (Photo: Bertil Akessons)

**Fig. 2:**
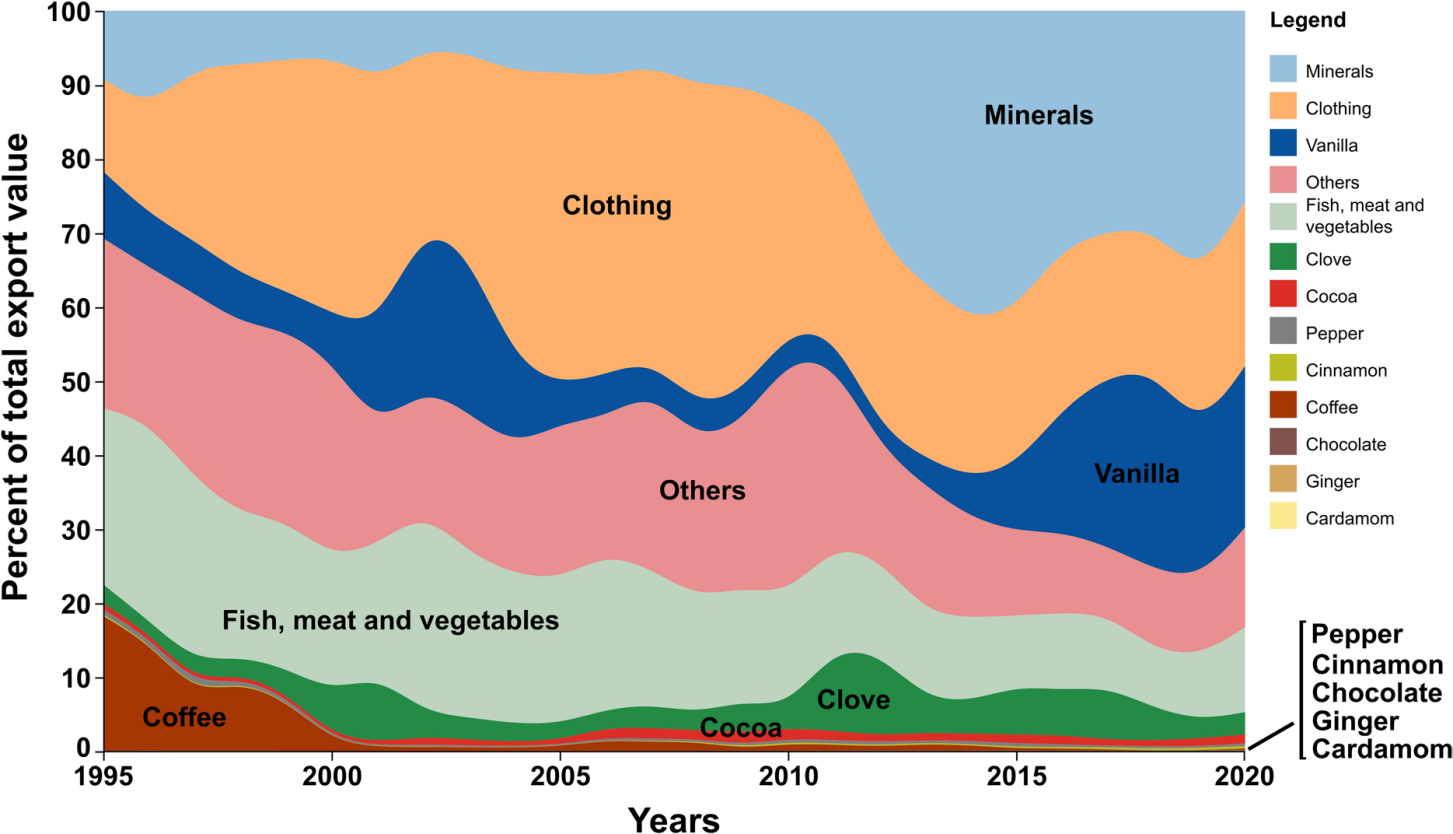
Annual contribution of agroforestry crops and other major export categories to total export value of Madagascar in percentage, for the period 1995–2020 (Gaulier and Zignago 2010). On average, agroforestry crops made up 27.4% of exports between 2015–2020. Note that both, overall categories, and the list of agroforestry crops at the bottom right corner, are ordered by decreasing share of export value in 2020. Figure created with RAWGraphs (Mauri et al. 2017)

To fill in this gap, we, first, provide an historic overview of agroforestry systems in Madagascar. For this historical overview and subsequent sections, we focus on the crops that have traditionally represented a major foundation of the Malagasy economy (i.e., vanilla, clove, coffee, and cocoa; Fig. 1). Second, through a systematic review of the literature, we present the current (from 2003 to 2022) state and extent of agroforestry in Madagascar (Fig. 2), including the main challenges encountered in agroforestry management and marketing, as well as the socio-cultural factors underpinning its prevalence in diverse regions of Madagascar. Finally, we use the knowledge brought together from past and present research to develop a vision for the future of agroforestry in the country.

## Methods

### Historical overview

**Fig. 3:**
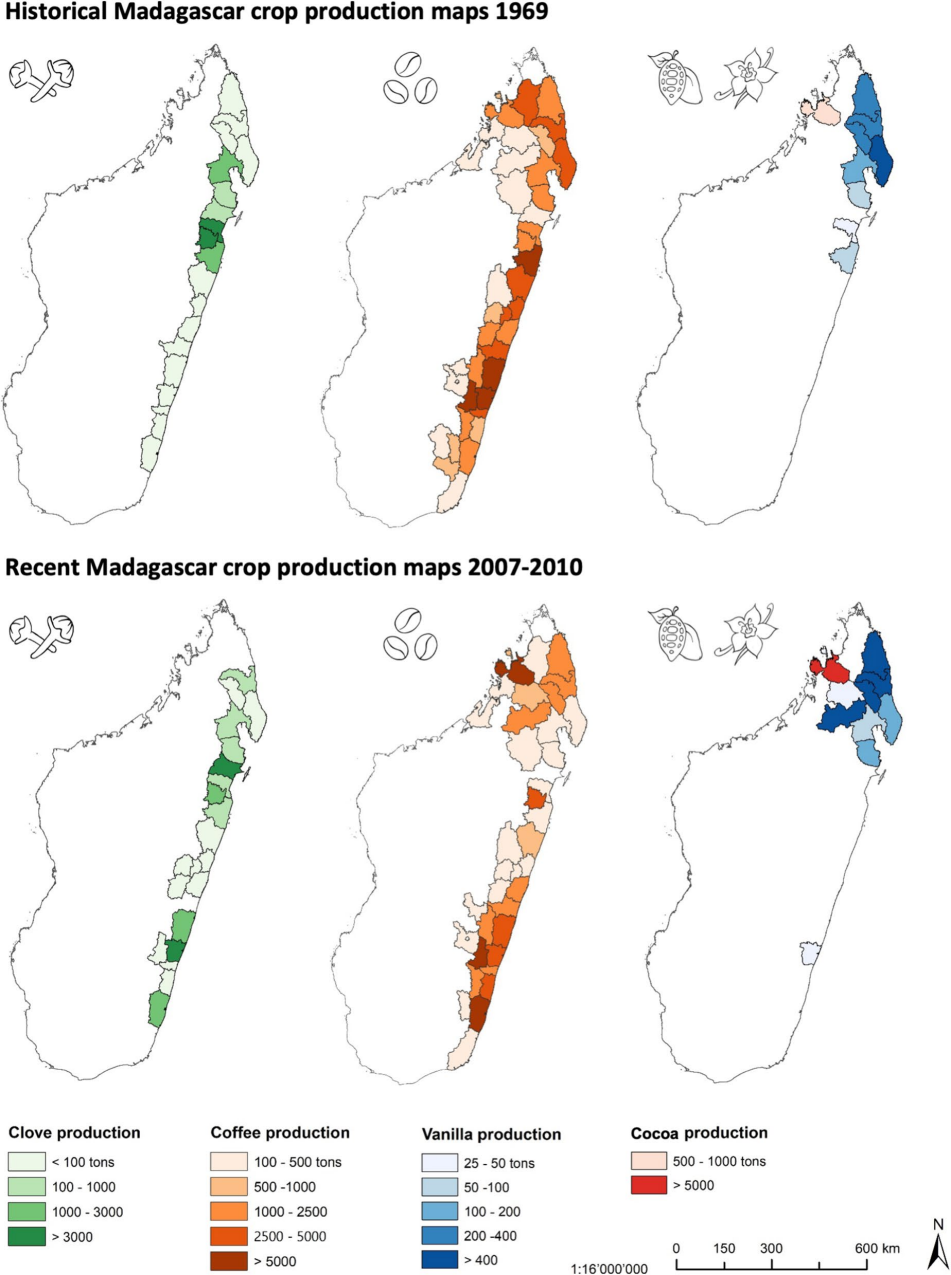
District-level production data for the four most important agroforestry-farmed cash crops clove, coffee, vanilla, and cocoa in Madagascar for the years 1969 (top row) and 2007–2010 (bottom row, average production across four years). Districts without recorded production of the respective crop are in white and their district boundaries are not displayed. Data source 1969: Le Bourdiec et al. (1969). Data source 2007–2010: MinAgri (2009, 2012)

To develop the historical overview of agroforestry in Madagascar, we compiled information from three sources. First, from online collections providing access to historical and colonial scientific journals not accessible through traditional bibliographic databases. We concretely looked into Mada Revues (Mada Revues 2021), the website for Malagasy scientific journals, in NumBA (Cirad 2021), the digital library in tropical agronomy of the CIRAD (Centre of International Cooperation in Agronomic Research for Development, by its French acronym), and in Persée (2021), a digital open access library established by the French Education Ministry. Even though these platforms provide several hundred records mentioning ‘agroforestry’ or crops that can be grown in agroforestry regimes, we restricted ourselves to using the items most relevant for providing a general overview of the history of agroforestry in Madagascar. These platforms, however, constitute a recommendable source of historical agroforestry literature to tap on for interested readers. Second, we retrieved information from some seminal geography and agronomy works, such as the 1969’s Atlas of Madagascar (Le Bourdiec et al. 1969), or compilations of agricultural research in former French colonial tropical Africa (Tourte 2005, volumes I to VI). And third, we compiled information on production, area and yields from 1961 to 2019 for the main agroforestry crops in Madagascar from the *FAO* (FAOSTAT) and obtained the contribution of different agroforestry crops to the total export value of Madagascar from 1995 to 2020 from *BACI: International Trade Database* (Gaulier and Zignago 2010). To elaborate the production maps presented in the study (Fig. 3), we digitized the historical maps from the 1969 Atlas of Madagascar (Le Bourdiec et al. 1969) and created 2007–2010 production maps using different regional agricultural productivity estimates provided by the agricultural statistic services from the Malagasy Ministry of Agriculture.

### Systematic review of current state of agroforestry

To systematically review literature on agroforestry in Madagascar, we used three different searches. We conducted all searches in both English and French and searched the databases covered by Web of Science (Core collection, subscription of the University of Bern). We did not restrict the type of research item nor the date of publication.

First, we used a search string encompassing agroforestry and Madagascar on 23.11.2021. This resulted in 99 literature items. Two authors, PL and DAM, then screened all 99 items on the abstract level. For 30 items, both decided to include them while for 46 items, both decided to exclude them. For 23 items, the two authors made contradicting decisions (76.8% agreement). Both then discussed each one of the items and jointly decided to include 15 and exclude 5. Therefore, we included 45 items to conduct a full-text screen.

Secondly, to capture items on agroforestry, crop cultivation, or marketisation that did not explicitly refer to agroforestry, we compiled a list of perennial crops mentioned in the context of agroforestry within the 45 reviewed items. The list contained 13 crops: coffee, banana, baobab, clove, vanilla, breadfruit, mango, jackfruit, cocoa, *tsiperifery*, lychee, avocado, and coconut (all in French and English, including multiple spellings, see Table S1). We conducted the search on 31.03.2022, combining each crop with “Madagascar”.

Thirdly, to capture items on crops that have so far not been mentioned in combination with agroforestry in Madagascar but that might hold potential for integration in agroforestry systems, we compiled a list of multi-annual crops with potential for integration in agroforestry contexts in Madagascar. The list contained 10 crops: cashew, cinnamon, rubber, jatropha, pepper, ginger, khat, litchi, citrus, ylang ylang (all in French and English, including multiple spellings, see Table S1). We conducted the search on 31.03.2022, combining each crop with “Madagascar”.

The second and third search resulted in 312 and 99 items, respectively. 19 items were included twice. Two authors, PL and DAM, then screened the remaining 392 items on the abstract level. For 37 items, both decided to include them, while for 335 items, both decided to exclude them. For 20 items, the two assessors made contradicting decisions (94.9% agreement). Both then discussed each of the conflicting items and jointly decided to include 12 and exclude 8. This resulted in an additional 49 items for full-text review, of which 18 were already covered by the first search. We therefore assessed 76 items on a full-text level.

**Fig. 4:**
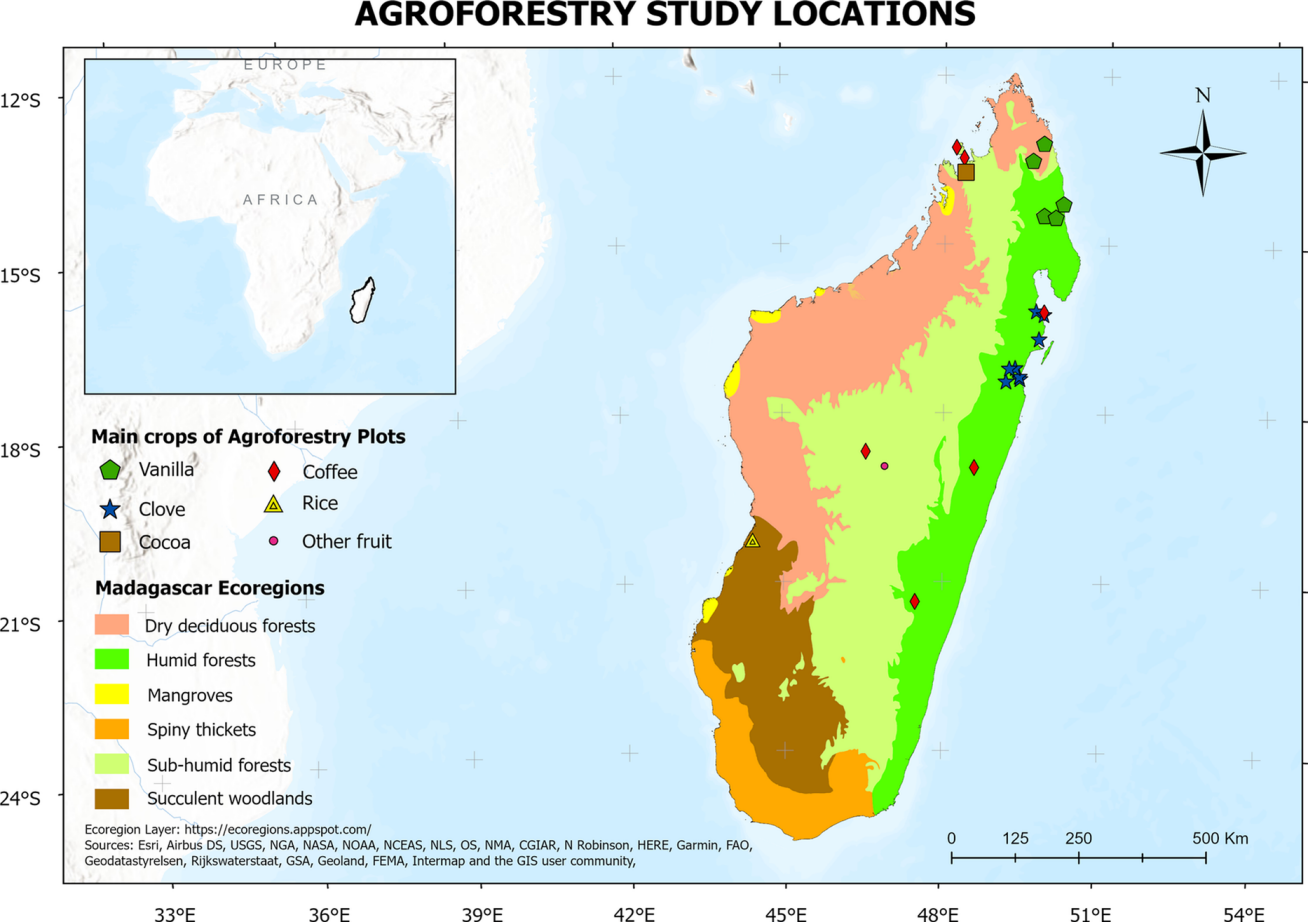
Map of Madagascar (right) with ecoregions and locations of studies on agroforestry (*N* = 30) included in the review with symbols representing crops. Location of Madagascar off East Africa (top left)

Co-authors PL and DAM screened full texts of 76 items in a systematic manner, excluding 46 items (41 because they did not focus on agroforestry, four because they did not focus on Madagascar, and one that was not a scientific article; see Fig. S1 for exclusion reasons and details). We then reviewed the remaining 30 items and collated information on study type, field of study, agroforest composition, land-use history and outcomes, factors promoting and inhibiting agroforest establishment, post harvest operations, and study location. We used this information throughout this paper, visualised certain aspects in Fig. 4, and provided the full scoring sheet as Supplementary Material (Table S2).

## Past

### Crop introduction

Agroforestry crops have historically represented a major component of the Malagasy economy (Danthu et al. 2014; Grisoni and Nany 2021). Despite their contribution to export earnings, their share decreased in favour of minerals and clothing since the turn of the millennium (Fig. 1). Like most other crops, all major agroforestry crops were introduced to Madagascar, in different waves (Haudricourt 1948; Beaujard 2011). First arriving were coconut (*Cocos nucifera*), banana (*Musa paradisiaca*), shampoo ginger (*Zingiber zerumbet*), and turmeric (*Curcuma longa*), brought in by Indonesian people (Tourte 2005, vol I; Gunn et al. 2011; Beaujard 2011). Citrus (Tourte 2005, vol I), and also likely the pomegranate (*Punica granatum*) were introduced later (Haudricourt 1948; Tourte 2005, vol II; Tourte 2005, vol III). Before the nineteenth century, the cashew (*Anacardium occidentale*) arrived, either from India or Brazil (Tourte 2005, vol III). Still later, a rapid wave of arrivals took place between 1800 and 1803, with several fruit trees being introduced through the work of André Michaux, founder of the short-lived experimental station of Ivondrona (Tourte 2005, vol III). Among these, notable examples are mango (*Mangifera indica*; Tourte 2005 vol II), guava (*Psidium Guajava*), and avocado (*Persea americana*).

However, the agroforestry crops that eventually contributed most to the Malagasy economy—clove, vanilla, coffee, and cocoa—only arrived later. The clove tree (*Syzygium aromaticum*) was first introduced on Sainte-Marie Island off eastern Madagascar in the 1820s from the Mascarene Islands (Ramanantsoavina 1971; Cocoual and Danthu 2018). A late arrival was also vanilla (*Vanilla planifolia*), introduced towards 1870 from Central America via metropolitan France and Reunion Island (Bouriquet 1946), around the same time as another Malagasy flagship export crop, the lychee tree (*Litchi sinensis*) (Tourte 2005, vol III). Cultivated coffee (*Coffea arabica*), was introduced in the east coast of Madagascar by Mascarene merchants, probably in the second half of the nineteenth century, even though wild coffee plants with low caffeine content were known in Madagascar since the late eighteenth century (Ramanantsoavina 1971). Finally, cocoa (*Theobroma cacao*), was the last major agroforestry crop arriving in Madagascar, introduced also in the east coast in the 1880s by creoles from Reunion Island (Streak 1996; Clarence-Smith 2000; Li et al. 2021).

During the French rule (1886–1960), production of crops that can be grown in agroforestry systems was strongly encouraged by colonial authorities to promote a positive trade balance for the island, and to counteract competition from the British colonies producing some of the same crops (Cocoual and Danthu 2018). The east coast experienced a substantial extension of plantations due to favourable climatic and environmental conditions (Moreuil 1971). During colonial times, French companies, such as the *Compagnie de Marseille*, were granted concessions for large-scale plantations (between 100 and 800 ha). These typically hosted several crops at the same time, likely in a mosaic of monocultures. However, plantations for specific crops were also known, such as clove on Sainte-Marie Island (Cocoual and Danthu 2018). Such focus on export crops during the colonial era had, however, important socio-economic and environmental implications, for example, forced labour from the prison population (Cocoual and Danthu 2018). The promotion of export crops also led to food shortages due to decreased subsistence farming, particularly on the East coast, which did become increasingly dependent on other regions for assuring food security (Jarosz 1994). Deforestation was also an outcome of the focus on export crops, derived from the need for firewood to fuel essential oil production units, such as for clove oil (Cocoual and Danthu 2018). Forests were also directly converted to plantations, which also led to increased soil erosion, especially on coffee plantations (Jarosz 1996).

In the remainder of this section we focus on the four agroforestry crops that either currently or historically have contributed most to Madagascar’s exports, vanilla, coffee, clove, and cocoa (Fig. 1), before providing some insights on a few other niche agroforestry crops.

### Vanilla: largest global producer in a fluctuating market

Vanilla is probably the most known Malagasy export product, and the island’s flagship agroforestry crop. Even though much of Madagascar’s east coast is suitable for vanilla cultivation (Bouriquet 1946), most production eventually took hold in the northeastern SAVA region. Secondary production centers are the Maroantsetra district, further south in the northeast, and the northwest, around Ambanja and on Nosy Bé Island (Fig. 3; MinAgri 2009, 2012). However, soon after intensive production started taking hold in Madagascar, Fusariosis, a disease caused by the soil fungus *Fusarium oxysporum*, began attacking plantations (Grisoni and Nany 2021). Losses of up to a quarter of the annual production triggered the launch of a breeding program to develop variants resistant to the disease in the early 1950s. The program, which lasted nearly 50 years, was eventually successful in developing two vanilla variants with increased resistance to Fusariosis and high vanillin content, leading to release for production in 1995 (Grisoni and Nany 2021).

Even though just a ton of vanilla was produced in Madagascar on the eve of the French colonial regime in 1896 (Bouriquet 1946), yearly exports were already nearly 900 tons between 1929 and 1938 (Correll 1953). From the 1920s through the 1980s, Madagascar was the world’s primary producer of vanilla, with some 1400 tons of average yearly production (FAOSTAT). From 1961, several mechanisms were introduced to buffer producer prices against global price fluctuation, and to provide order to the national vanilla sector, and stability to the global vanilla market (Cadot et al. 2006). Specially relevant were the Vanilla Stabilization Fund, and the Vanilla Alliance between Madagascar, Comoros and Reunion Island, which helped Madagascar retain its hegemony on the global market through the 1980s while maintaining high global prices (Maret 2007; Blarel and Dolinsky 1995). Encouraged by high vanilla prices, Indonesia, the second largest producer, expanded vanilla cultivation in those decades, leading to increasing exports reaching 45% of the global market in the second half of the 1980s (Blarel and Dolinsky 1995; Brown 2009). Simultaneously, Madagascar experienced a drop in the world market share to just 40% by the early 1990s (Blarel and Dolinsky 1995). This evolution was not only due to Indonesia’s competition, but also due to increasing export taxes and the parallel decrease in prices received by farmers during the Malagasy Second Republic (1975–1992). This discouraged production, and the Stabilization Fund was eventually eliminated in 1995 (Cadot et al. 2006). By 2007, Madagascar had recovered much of the world market, with its sales accounting for 60% of the global trade in vanilla (Brown 2009). In terms of contribution to the Malagasy economy through exports, vanilla went from representing only 9% of the total export value in 1995 (46 M USD), to a peak of nearly 27% in 2018 (nearly 940 M USD) under very high prices. Since then, this share has decreased alongside prices but remains at around 20%, contributing between 540 and 650 M USD annually (Gaulier and Zignago 2010).

### Coffee: from the most important export product to near-total collapse

Coffee has been traditionally produced in Madagascar along the east coast and in the island’s north and northwest, although production centers shifted in importance over time (Fig. 3). Coffee production had likely gained some importance already in the 1870s, including in the Central Highlands, before the French invasion (Ramilison 1985). However, in the late nineteenth century *C. arabica* was attacked by coffee leaf rust (*Hemileia vastatrix),* making the crop largely disappear (Ramanantsoavina 1971). In consequence, *C. Arabica* was replaced by *C. canephora,* both *robusta* and *kouilou* varieties, introduced around 1900 and 1905 (Ramanantsoavina 1971). Coffee production gained traction especially in the southern half of the east coast where, in the late nineteenth century, European settlers had already taken some of the best areas for production (Ramilison 1985). By the early 1970s, some 210,000 ha of *robusta-canephora* were cultivated (Fig. 3), alongside 7000 and 8000 ha of *arabica* (Ramanantsoavina 1971). This production was mainly achieved by smallholders, while industrial production amounted to just 15% of the national output. However, yields varied substantially across production systems, from 500–1200 kg/ha per year attained by industrial plantations, to 200–300 kg/ha obtained by smallholders, with even lower yields in old smallholder plantations which accounted for up to 50% of smallholder plantations (Ramanantsoavina 1971). In the peak of coffee cultivation in Madagascar in the early 1980s, production involved some 450,000 farmers (Ramilison 1985). Prominence of the crop faded thereafter, although in the early 2000s, between 350,000 and 400,000 households were thought to still produce the crop (World Bank 2003; LMC International 2000).

Regarding production and its export role, coffee evolution is of astonishing early growth, from just around 160 tons exported by 1912, to some 1400 in 1919, more than 10,000 in the early 1930s, and above 40,000 tons at the end of that decade (Ramilison 1985). At the time, Madagascar was the largest exporter of all French colonies in Africa (Blanc-Pamard and Ruf 1992). In the 1940s however, coffee production decreased due to two factors. First, the impact of the Second World War, which drove the abandonment of coffee production in favour of staple food. And second, the nationalist insurrection of 1947 against the French rule and its subsequent repression by the colonial authorities, which hit the producing areas along the east coast, leading to the abandonment of plantations (Ramilison 1985). Nonetheless in the 1950s, extension of plantations and introduction of measures to improve coffee cultivation (e.g., extension services, phytosanitary efforts, and special funds) led to increased production, with around 50,000 tons exported yearly by the end of the decade (Ramilison 1985). However, a bad international price conjuncture and a series of cyclones in 1959 (Aldegheri 1959; Le Monde 1959) and further disasters in 1961 brought new problems to production, especially for smallholders (Ramilison 1985). A Stabilization Fund was also created for coffee in 1961, again to buffer the price received by producers from external fluctuations, and to centralize coffee transport from production areas, and subsequently export it (Maret 2007; World Bank 1984). The following year, the International Coffee Agreement was signed, although with Madagascar in a relatively unfavorable position, getting an export quota of 49,730 tons, or less than 2% of the global trade regulated by the agreement (Ramilison 1985). With the intention of increasing production and standing in the international scene, Madagascar launched an extension programme called *Operation Café* along the east coast in 1966 (Ramilison 1985). The *Operation Café* would be relatively successful, and Madagascar would be producing above 80,000 tons of coffee most years in the 1970s while exporting some 65,000 tons on average. These efforts, together with growing global coffee prices (World Bank 1984), led to an increase of coffee’s contribution to the overall export value from some 28% in 1972 to 53% in 1980. Together with clove and vanilla, coffee then represented two thirds of Madagascar’s source of foreign currency (Ramilison 1985).

However, in 1988 the Coffee Stabilization Fund was eliminated (LMC International 2000) as part of the structural adjustment programs imposed by the World Bank and the International Monetary Fund, which was lending money to Madagascar since 1980 (Rasolofo-Jaonarison 1998; Mukonoweshuro 1994). This resulted in a partial neglect of the coffee sector, while the government was focusing much of its efforts in supporting food crop production (LMC International 2000), and the vanishing of the role of coffee as a cash crop up to present time, as it was unable to provide income for farmers (Danthu et al. 2014). In recent decades, and even if still producing above 50,000 tons/year (FAOSTAT), Madagascar passed from exporting some 40,000 in the mid 1990s, to below 5000 tons two decades later (Gaulier and Zignago 2010), mostly due to decreasing coffee quality resulting from aging plantations, lack of maintenance, limited access to inputs for producers, and high incidence of diseases (LMC International 2000; World Bank 2003). Today, most of the production is consumed nationally (Danthu et al. 2014). Thus, coffee went from representing around 18% of the total export value in Madagascar in 1995 (some 93 M USD), to just 0.15% in 2020 (or some 4 M USD), below what is contributed by pepper or cinnamon (Gaulier and Zignago 2010).

### Clove: steady increase in production and sales of a niche crop

Even if being the earliest major agroforestry crop introduced to Madagascar, and some exports already reported in the first half of the nineteenth century (Campbell 1991), production of clove only expanded substantially from 1895 until 1958 (Ramanantsoavina 1971). This expansion happened in several waves, first on Sainte-Marie Island, and then on the mainland, particularly the Analanjirofo region (aptly, “Clove Forest'' in Malagasy), which remains the main producing area (Danthu et al. 2014). The first distillation workshops for clove essential oil, for which Madagascar is the largest world producer (Danthu et al. 2014), appeared somewhere in the first decade of the twentieth century on Sainte-Marie and in 1923 on the east coast. Authorisations to operate these workshops were first granted only to European settlers, and later to Malagasy, especially from 1945 onwards (Cocoual and Danthu 2018). From the mid twentieth century, and after the Second World War, Madagascar’s clove production experienced a further boost, especially as consumption of *kretek* cigarettes, which use clove flavour, took off in Indonesia. Particularly important in this regard was the trade agreement signed between the two countries in 1951, allowing Madagascar to export clove to the Asian country. This was followed by further export agreements with Poland, Czechoslovakia, and Austria, among other countries (Cocoual and Danthu 2018). Also, as for vanilla and coffee, a Stabilization Fund to smoothen clove price fluctuations for producers and centralize marketing production was established in 1961 (World Bank 1984), which also supported extension of plantations and research for enhancing production through the 1960s and 1970s (Ranoarisoa 2012; Danthu et al. 2014). However, the 1980s witnessed a series of adverse developments for clove. First, Indonesia, a major importer from Madagascar, temporarily stopped imports after attaining self-sufficiency (Danthu et al. 2014). Second, the Clove Stabilization Fund was eliminated in 1988 (Ravelosoa et al. 2015), and most measures taken by the colonial administration were abandoned. However, the 1990s saw a decline of clove exports from Zanzibar, Madagascar’s first competitor in the global export market, which allowed Madagascar to become the world’s first exporter until present. This resurgence was further fueled by the coffee crisis that drove producers to switch to clove as source of income (Danthu et al. 2014). This situation, however, coincided with declining prices for clove, paralleled with increasing ones for clove oil, which encouraged production of the latter in the last decades of the twentieth century (Ranoarisoa 2012).

Regarding clove production quantities, numbers expanded from 34 tons/year at the beginning of the twentieth century (Ranoarisoa 2012), 1600 tons/year in the late 1920s (Danthu et al. 2014), some 6000 tons/year through the 1960s, around 10,000/year tons in the 1970s and 1980s, some 13,000 tons/year in the 1990s and 2000s, and increase to some 20,000 tons in the 2010s (FAOSTAT). In terms of exports, clove has enjoyed an increasing share of the total export value, reaching a maximum of just above 11% of contribution in the early 2010s (Fig. 1). Quantities exported fluctuate strongly given that clove trees do not produce every year (Danthu et al. 2014), and has remained around 15,000 tons/year from 1995 to 2020, although passed from contributing some 13 M USD in 1995, to some 74 M USD in 2020, down from a peak of 233 M USD in 2017 (Gaulier and Zignago 2010; Fig. 1).

### Cocoa: regionally important in the northwest

Cocoa was the last major agroforestry crop introduced to Madagascar. Production is relatively small compared to other major producing countries but its quality is highly considered globally (Li et al. 2021). After introduction in the 1880s, cocoa production started growing rapidly, with between 5000 and 6000 trees already planted by 1883 (Campbell 1996). The 1883–1885 Franco-Merina war led to the temporary abandonment of cocoa plantations, although production resumed after the conflict, with expansion encouraged by the spread of coffee leaf rust on *Arabica* coffee in the 1880s, as farmers were prompted to try crops alternative to coffee. By the end of the 1880s, more than 150,000 cocoa trees were planted along the east coast, with the frontier moving north and south from Toamasina since 1888 (Campbell 1996), and even a modest boom taking place in the 1890s (Streak 1996). However, the northwest region of Sambirano around Ambanja would start gaining in importance for cocoa production, given that, in contrast with the east coast, provided shelter from destructive tropical cyclones and storms, had less pests, and crucially, enjoyed a dry season that allowed to dry the cocoa harvest (Clarence-Smith 2000:134). Until 1923, cocoa production was dominated by creoles cultivating it on the east coast, but would move northwards and start being more dominated by Malagasy producers for three reasons. First, the lack of capital of creoles producers, second, the high costs of labour on the east coast, and third, the frequency of the storms in this region (Streak 1996). Eventually, cultivation of cocoa took hold in the Sambirano plain, which remains the main producing region on the island until present (Fig. 3).

Regarding cocoa production and export, Madagascar slowly increased both during the first half of the twentieth century, from 28 tons exported in 1910 (Li et al. 2021), to a production of between 300 and 400 tons in the early 1960s. This was produced on some 1000 ha (Destrez 1960), with expansion by Malagasy farmers further promoted by the government during that decade (Streak 1996). Production was also encouraged by the privileged access granted to the metropolitan market by the French government, both before and after independence (Streak 1996). Although cocoa production increased rapidly in the 1970s, reaching some 6000 ha cultivated producing some 1700 tons annually by the end of the decade (FAOSTAT), the contribution of Malagasy cocoa to global production represented just a 0.12% in 1979 (Streak 1996). Despite a steady increase in production on the island up to present time (FAOSTAT), cocoa is yet to take off as a major contributor to the overall economy of Madagascar. By 2017 Madagascar was producing some 6000 tons on some 24,000 ha in the Sambirano area, which provides 95% of the national production involving both smallholders and industrial plantations (Le Bellec et al. 2017). In terms of export value, cocoa went from representing some 4 M USD in 1995, to around 30 M USD in 2020, hovering around a contribution of 1% total Malagasy export value in the 2010s (Gaulier and Zignago 2010, Fig. 1).

### Other products: exploring the potential of niche crops and spices

A number of other agroforestry crops have some economic importance in Madagascar in addition to the four just reviewed. For example, Madagascar is the third largest producer of lychee (*Nephelium litchi*) globally, after China and India, with a current production of some 100,000 tons, mostly produced around Toamasina and to a lesser extent around Mananjary (Business France 2018). Even though only 25% of the production is exported (Business France 2018), Madagascar is Europe’s main supplier, passing from just some 500 tons exported in the mid 1980s to nearly 9000 tons just ten years later (Loelliet 1994) and some 20,000 tons during the 2010s (Jahiel et al. 2014). Pepper (*Piper nigrum*), introduced in Madagascar in 1900 (Ramanantsoavina 1972) and often cultivated together with coffee (World Bank 2003) or cocoa (Fig. 3), is also of some importance for Madagascar’s economy, contributing on average 9 M USD a year in the 2010s (Gaulier and Zignago 2010). Even though quantities exported have increased from 1300 tons/year in 1995–2010 to 3500 tons in 2018, its contribution to the export value actually peaked in 1997 at 1%, remaining below 0.5% in the 2010s (Gaulier and Zignago 2010).

Cashew (*Anacardium occidentale*) production has a long tradition in Madagascar (Lefèbvre 1969), with average yearly production increasing some 1000 tons per decade since the 1960s, or from some 2000 tons/year in the 1960s, to some 7000 tons/year in the 2010s (FAOSTAT). Even if Madagascar exports some 10,000 tons/year currently (L'Express de Madagascar 2022a), mostly from the main producing area around Ambilobe in the north, its contribution to the export value is so far negligible (Gaulier and Zignago 2010). However, cashew production has been proposed as an option to provide alternative livelihoods to local communities around protected areas (Durbin and Ralambo 1994; Gardner et al. 2013).

Madagascar also produces cinnamon, especially of the *Cinnamomum verum* species (Lallemand et al. 2000), which is farmed by some 2500 farmers predominantly on the east coast (CTHT 2023). Overall, 2500 tons a year were exported in the late 2010s with a value of 90 M USD (Gaulier and Zignago 2010). Even barely known, Madagascar also produced and exported rubber (*Hevea brasiliensis*) between 1891 and 1914 and again during the Second World War (Danthu et al. 2016). However, its production history was cut short by the competition from Asian countries, and the perceived impacts of its unsustainable exploitation, which, in unexpected events, triggered the creation of the first protected areas in the country (Danthu et al. 2016). Also worth mentioning is the Malagasy wild pepper, the *tsiperifery*, which has so far a limited contribution to the economy despite its potential for domestication (Ceccarelli et al. 2021). Madagascar also grows oil palm on some 7000 ha (Selina Wamucii 2023), of which the large majority is consumed nationally, with just some 500 tons exported on an average year through the 2010s (OEC 2021). While currently planted in monocultures, oil palm could also form part of agroforestry systems (Zemp et al. 2023). Lastly, several plant species are harvested for essential oils, for example ginger, cinnamon, clove, and eucalyptus (Koroch et al. 2007).

## Present

Currently, most production for crops that can be produced under agroforestry is carried out by smallholders. This involves some 70,000 farmers in the vanilla sector in the SAVA region alone (Tridge 2020), some 30,000 in clove production (AFS4Food 2012), another 30,000 farmers in lychee farming (Jahiel et al. 2014), some 380,000 in coffee (United Nations 2018), and around 30,000 in cocoa (Kentsop Suayo 2017), leading to a lower estimate of 500,000 farmers being involved in producing such crops. The plantation regime prevalent during colonial times for the main export crops that can be produced under agroforestry in Madagascar has given place to relatively complex agroforestry systems (Arimalala et al. 2018; Bing 2015; Wurz et al. 2022). In some cases, production is carried out under contract farming schemes, such as in part for the cases of vanilla (Andriamparany et al. 2021; Blum 2021) and cocoa (Callahan 2019), or cooperatives, as for sections of the vanilla (Ralandison 2021), coffee (L'Express de Madagascar 2022b), and cocoa sector (Ethiquable 2023).

### Current state and extent of agroforestry research

**Fig. 5:**
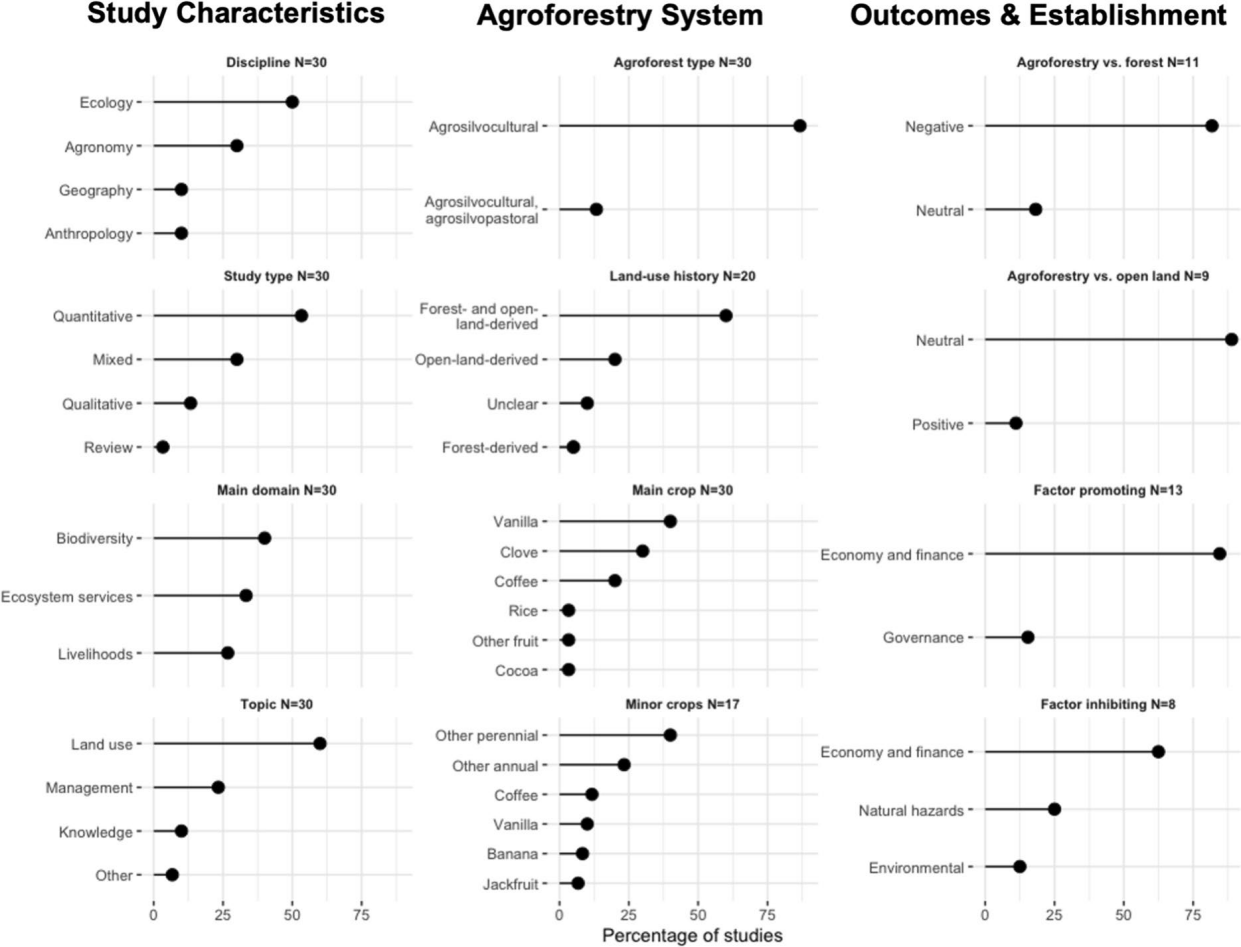
Percentage of studies on agroforestry in Madagascar (*N* = 30) that have specific study characteristics (left column), that focus on certain types of agroforestry systems and crops (middle column), and that study various factors determining agroforestry outcomes and establishment

We found 31 studies on agroforestry in Madagascar using our definition of agroforestry (see introduction) and our inclusion criteria (see methods). Available research is geographically biased towards the northeast of the country (Fig. 4), the region where most cash crops are grown (Minten and Barrett 2008). We further identify a major bias towards studies on agroforestry systems focusing on certain cash crops, namely vanilla and clove, that are more prominently studied than others, namely coffee and cocoa. This is despite comparable production volumes (Fig. 3). These biases highlight that there is a risk of missing out on development and conservation potential of less well known agroforestry systems elsewhere in Madagascar. Studies are furthermore predominantly from the field of ecology, use quantitative approaches, focus on land use, and investigate biodiversity and ecosystem services more commonly than livelihood implications (Fig. 5).

### Characterizing agroforestry systems in Madagascar

The majority of studied agroforestry systems are agrisilvicultural, combining trees with crops (Fig. 5). However, four reviewed papers focus on both agrisilvicultural and agrosilvopastoral systems, in particular clove trees combined with cattle grazing. Agroforestry systems also differ between the island's many ecoregions. Cloves and vanilla are the two principal crops grown in the northeast. Coffee and cocoa are grown in Madagascar's eastern and northwestern regions, respectively. All systems, either originate from forests or open land, with key implications for their conservation value (Martin et al. 2020). Outcomes for agroforestry systems having vanilla, clove, and cocoa as a main crop range from neutral to positive. Common crops associated with these main crops are fruit trees, including jackfruit, avocado, and mango, as well as banana and coconut. In the western region of Madagascar, an agroforestry system combining rice, maize, and cassava with trees has been investigated.

### Biodiversity in agroforestry systems in Madagascar

Biodiversity and ecosystem service outcomes of agroforestry in Madagascar are increasingly studied in relation to both open land (e.g., fallow land, annual crops) and forest. When comparing biodiversity in agroforestry systems to open land, those agroforests established inside forests (forest-derived) often outperform open land, while agroforests established on open land (open-land-derived) are comparable or slightly more biodiverse than open land (Martin et al. 2022). When comparing biodiversity in agroforestry systems to forest, research has shown that agroforests often fail to upkeep (when forest-derived) or restore (when open-land-derived) biodiversity to levels comparable to old-growth forest (Hending et al. 2018; Martin et al. 2022). Nonetheless, forest-derived agroforests may be important to retain relatively high levels of biodiversity—on par with heavily used forest fragments—in agricultural landscapes (Martin et al. 2022).

These findings stem from studies across multiple crops (cocoa (Webber et al. 2019); vanilla (Martin et al. 2022); coffee (Blumgart et al. 2017)) and taxa (bats (Hending et al. 2023); lemurs (Hending et al. 2018; Webber et al. 2019); birds (Martin et al. 2021a, b); amphibians and reptiles (Blumgart et al. 2017; Fulgence et al. 2022); butterflies (Wurz et al. 2022); ants (Rakotomalala et al. 2021); trees (Osen et al. 2021); herbaceous plants (Raveloaritiana et al. 2021) while certain agroforestry systems, like clove, and certain taxa, like small mammals and most insects, are not studied yet. However, meta research on the importance of land-use history for biodiversity in tropical agroforestry systems suggests that above-listed results may hold for different taxa and crops (Maney et al. 2022; Martin et al. 2020).

### Ecosystem services in agroforestry systems in Madagascar

For ecosystem services, the results are more complex. Agroforests are less important for many regulating ecosystem services than forests, but likely outperform open land, for example fallow land. This is the case for water regulation (Raveloaritiana et al. 2023; Zaehringer et al. 2017), pest control (Schwab et al. 2021), and carbon stocks (Soazafy et al. 2021). Similarly, smallholder farmers practicing agroforestry in the central highlands of Madagascar have smaller carbon footprints than those not implementing such practices (Rakotovao et al. 2017). Agroforests supply provisioning ecosystem services such as firewood, timber, and fruits even when focusing on cash crop production (Mariel et al. 2021a, b; Raveloaritiana et al. 2023; Zaehringer et al. 2017). Few papers assessed yields in agroforestry systems. For vanilla, yields did not differ between forest- and open-land-derived agroforests, nor along shade or precipitation gradients (Martin et al. 2021a, b)**.** Instead, agroforests with higher planting density and older vanilla vines had higher yields (Martin et al. 2021a, b). Another paper from the same region but from other agroforests found positive effects of pollination labor input, planting density, and vine length—but not vein age and other predictor variables—on vanilla yields (Wurz et al. 2022). For cultural ecosystem services, studies are limited but agroforests have been identified as important sites for ceremonies and gatherings (Osterhoudt 2010)**.**

A key limitation is that most studies do not compare households with (or with more) agroforestry to households without (or with less) agroforestry. Furthermore, none of the studies we found directly linked agroforestry practices to ecosystem services and further to agriculture production within agroforestry systems. Stakeholder demand for services and potential mismatches between ecosystem service demand and supply have also not been studied. Overall, we find geographical and crop-specific biases in this body of literature, with studies focusing on cash crops, especially clove and vanilla, in the northeast of Madagascar.

### Socioeconomic and environmental factors enabling and hindering agroforestry adoption

Agroforestry is considered a way for Malagasy people to earn a living, helping them to become more resilient and recover from challenges (Osterhoudt 2018; Herrera 2021; Danthu et al. 2014; Martin et al. 2021b). People think of it not only as a means to earn income (Mariel et al. 2021a, b), but also as something that brings the community together and preserves their shared history (Osterhoudt 2018). Moreover, agroforestry is used to establish ownership of land still governed by traditional laws in specific parts of Madagascar (Evans et al. 2020). In areas where agroforestry is widely adopted, it assists local communities in attracting markets and economic opportunities (Nambena et al. 2003).

Nevertheless, there are obstacles discouraging farmers from adopting agroforestry. These include difficulties in transporting agroforestry goods to markets, insufficient capital for investment, ongoing poverty, and volatile global market prices (Pfund et al. 2011; Tsujimoto 2014). Studies also point out challenges such as finding suitable land to initiate an agroforestry system (Nambena et al. 2003), political instability, changes in land ownership rules, and a lack of legal awareness (Evans et al. 2020). In certain regions, agroforestry practitioners are facing additional challenges posed by natural hazards. Cyclones annually inflict significant damage on human lives, infrastructure, and farmlands in eastern Madagascar (Danthu et al. 2014; Osterhoudt 2018; Michel et al. 2021), further exacerbating socio-economic struggles (Llopis 2018). The effect of cyclones is notably highlighted in studies focusing on agroforestry crops like vanilla and cloves (Brown 2009; Llopis et al. 2019).

## Discussion: potential future for agroforestry in Madagascar

Locally called ‘ala vadim-boly’ ("forest with agricultural land") or more broadly ‘Tanimboly’ (“cultivated land”), agroforestry systems in Madagascar center around various cash crops for local consumption or export, particularly vanilla, coffee, cocoa, and clove, mixed with various fruit trees like banana, papaya, jackfruit or litchi, along with wood trees such as *Grevillea* spp. or *Albizzia* spp. As shown in this review, agroforestry crops have been present in Madagascar at least since the early nineteenth century (Tourte 2005 vol III; Ramanantsoavina 1971). Many of the crops sold for export were actively introduced and promoted by external actors, with the French colonial power playing a major role in mainstreaming key cash crops (Rabearimanana 1985; Isnard 1951). Between 2015 and 2020, crops that can be farmed in agroforestry systems contributed 27.4% to Malagasy exports (Gaulier and Zignago 2010). Here we argue that there is great potential for agroforestry to contribute to sustainable development in Madagascar and outline factors that may determine whether this potential will be realized.

### Maintaining existing agroforestry systems

Going through the websites of local or international NGOs working in Madagascar, agroforestry is promoted as an activity for (1) improving household income and food security; (2) restoring degraded lands, increasing water provision and conserving biodiversity: (3) enhancing resilience to climate change. For this, they aim to practice sustainable agriculture by combining the concept of agroforestry and permaculture in community projects such as school gardens (vegetables plants and fruits trees), demonstration plots (medicinal plants, ginger and silkworm host trees).

Indeed, agroforestry systems already play a major and increasing role in smallholder agricultural landscapes (Llopis et al. 2019; Mariel et al. 2023), particularly in humid eastern Madagascar (Fig. 3). Importantly, these systems are usually not the result of extension campaigns by external actors, but represent a traditional land use practiced for generations (Mariel et al. 2021a, b; Osterhoudt 2018). Maintaining those existing systems into the future is thus as important as promoting the extension of agroforestry to realize promised benefits, especially given repeated instances of external actors trying to promote transitions to ‘more efficient’ and less diverse cropping systems (Le Bellec 2017). Turning this around, private sector initiatives like sustainability certification (Blackman and Rivera 2011) could specifically reward the production in traditional agroforestry systems and support farmers in achieving higher yields or better labor productivity (see section "Production and marketing schemes" Production and marketing schemes).

### Promoting the extension of agroforestry systems

Agroforestry in Madagascar has the potential to benefit more regions, additional cropping systems, further land area, a greater number of people, additional ecosystem services, and a higher number of species than today. However, multiple factors should be considered to realize these benefits and various hurdles need to be overcome.

To overcome the geographic bias that agroforestry is predominantly practiced in the humid east, agroforestry needs to extend into additional crops that are currently little farmed or not farmed in agroforestry systems. Here, multiple crops hold potential, for example cashew, which is already cultivated in relatively dry parts of western and northwestern Madagascar (Zoma 2005). But also less widely farmed crops, also coined orphan crops (Tadele 2019), may hold potential. This is particularly the case since labor costs in Madagascar are low (Minten and Barrett 2008), making it possible to competitively farm labor-intensive crops if reliable value chains exist.

To extend agroforestry to more agricultural lands, agroforestry needs to be an attractive farming option for smallholder farmers. Building on our analysis of factors promoting and hindering agroforestry in Madagascar, we suggest that infrastructure, market access, and value chains are essential to market cash crops. Farmer training could also increase labor and per area productivity of existing and emerging agroforestry systems (Minten and Barrett 2008) while valuing traditional practices. In this context, combining cash with subsistence crops in diverse agroforestry systems further seems as a way forward to promote various sustainable development goals in concert.

To benefit a greater number of people, agroforestry also needs to be a land use option for the poorest households within communities. These typically face additional hurdles for adopting agroforestry, for example limited access to fertile land, insufficient land tenure, or initial investment barriers (Mercer 2004; Bettles et al. 2021). Overcoming these hurdles will require targeted actions by the state and NGOs which could have the potential to make agroforestry a viable land use for more people.

To promote additional ecosystem services and support more, and possibly more rare or threatened, species, agroforestry systems can further be optimized internally. For example, maintaining or increasing shade-tree canopy cover in vanilla agroforests benefits tree and reptile species richness and promotes endemic reptiles, ants, and herbaceous plants without negatively affecting yields (Wurz et al. 2022).

To limit possible negative consequences of agroforestry, several safeguards should be considered. On the social side, cash-crop focused agroforestry could result in high vulnerability of farmers if they are fully dependent on cash crop income for their livelihoods, especially under price fluctuations (Celio et al. 2023), theft risks (Neimark et al. 2021) and related insecurity (Llopis et al. 2022), and increasingly common extreme weather events (Brown 2009). On the environmental side, agroforestry should not be expanded at the cost of forests, neither by directly planting inside forest (Martin et al. 2020, 2022), nor by cutting forests for agroforestry. In this context, leakage (Meyfroidt et al. 2018) should also be considered: for example expanding agriculture on land previously used for shifting cultivation can only be considered a positive environmental outcome if shifting cultivation is not displaced into forests. Both social and environmental considerations highlight the importance of embedding agroforestry in a broader landscape context.

### Agroforestry as part of tree-rich multifunctional agricultural landscapes

Multifunctional agricultural landscapes provide a suite of essential ecosystem services, protect (agro-)biodiversity (Shennan-Farpón et al 2022), and enable resilient rural livelihoods (Kremen 2020). Importantly, such landscapes typically combine various cropping systems from extensive to intensive, with agroforestry making up an essential but not exclusive element. Alongside agroforestry and staple crop production, forest conservation for ecosystem services and biodiversity protection and the restoration of key wildlife corridors are also important. Hurdles to such landscapes in the Malagasy case are ongoing challenges in the protection of remaining forests (Jones et al. 2019), low agricultural productivity of the staple crop rice (Dröge et al. 2022), and difficulties in water management (Harifidy and Hiroshi 2022).

Within such multifunctional landscapes, agroforestry would be an essential enabling element of an urgently necessary forest transition in Madagascar by creating livelihood opportunities that do not rely on further forest clearance and by enhancing tree cover within agricultural landscapes.

### Production and marketing schemes

Multiple production and marketing schemes have been developed to increase the quality, quantity, or sales of agricultural products from agroforestry systems. For example, multiple contract farming schemes exist in vanilla farming (Blackman and Rivera 2011); Hänke et al. 2018; Blum 2021). Medium to large exporters typically organize farmers groups with the goal to achieve higher qualities, stable quantities, and less fluctuating prices. Many of these contract farming schemes are also linked to sustainability certification (e.g., Organic, Rainforest Alliance, Fair Trade), essentially since the assurance of the sustainability standards requires close multi-year relationships between buyers and exporters (Blum 2021). Some of those standards explicitly require a certain level of tree cover in cropping systems, thereby promoting the maintenance of traditional agroforestry (Blum 2021). However, benefits of such contractual arrangements for farmers may be mixed due to selection biases and higher production costs that may not necessarily be compensated by higher prices (Blum 2021).

If farmers would act together in cooperatives, they could benefit from collective action rewards such as improved market information, increased bargaining power, better labor management, or increased production (Gyau et al 2014). Also, such farmers cooperatives could make profit from turning their sequestered carbon into carbon credits (Telwala 2023). For example, some projects help smallholder farmers working with agroforestry by selling carbon credits to enterprises wishing to compensate for their emissions (Wollenberg et al 2022).

### Future research to inform agroforestry practice and promotion

Much research to date has focused on outcomes of agroforestry for biodiversity and ecosystem services while hardly any research concerns the factors inhibiting and promoting agroforestry adoption in Madagascar (see section "Socioeconomic and environmental factors enabling and hindering agroforestry adoption"). On top of addressing this bias, future research should focus on approaches to make agroforestry more resilient to shocks, investigate ways to diversify agroforests, and look into the potential of creating ‘novel’ agroforestry systems with crops currently not on the radar and in regions where agroforestry currently plays a minor role.

## Conclusion

Agroforestry already plays an important part in the daily lives of at least 500,000 Malagasy farmers, particularly on the humid eastern escarpment. Here, we have outlined the history of agroforestry, have taken stock on the state of agroforestry research, and have elaborated on the potential that agroforestry holds for the future in Madagascar. We have shown that multiple hurdles need to be overcome to make agroforestry an attractive land use option for smallholder farmers and to deliver multifunctional agricultural landscapes that work for people and nature. To achieve this, key gaps in policy development may need to be closed, bearing the question how Madagascar could catch up on the development of innovative agroforestry policies as pioneered in other countries such as India, Malaysia, or Tanzania?

### Supplementary Information

Below is the link to the electronic supplementary material.

Supplementary file1 (PDF 226 kb)

Supplementary file2 (CSV 163 kb)
