# Supplementary material for: Agroforestry in Madagascar: past, present, and future
Source: Agrofor Syst. 2024 May 10;98(6):1659–80. doi: 10.1007/s10457-024-00975-y (PMC11286679; doi:10.1007/s10457-024-00975-y)
Supplement: Supplementary file 1 — Supplementary file1 (PDF 226 kb) [file 10457_2024_975_MOESM1_ESM.pdf]

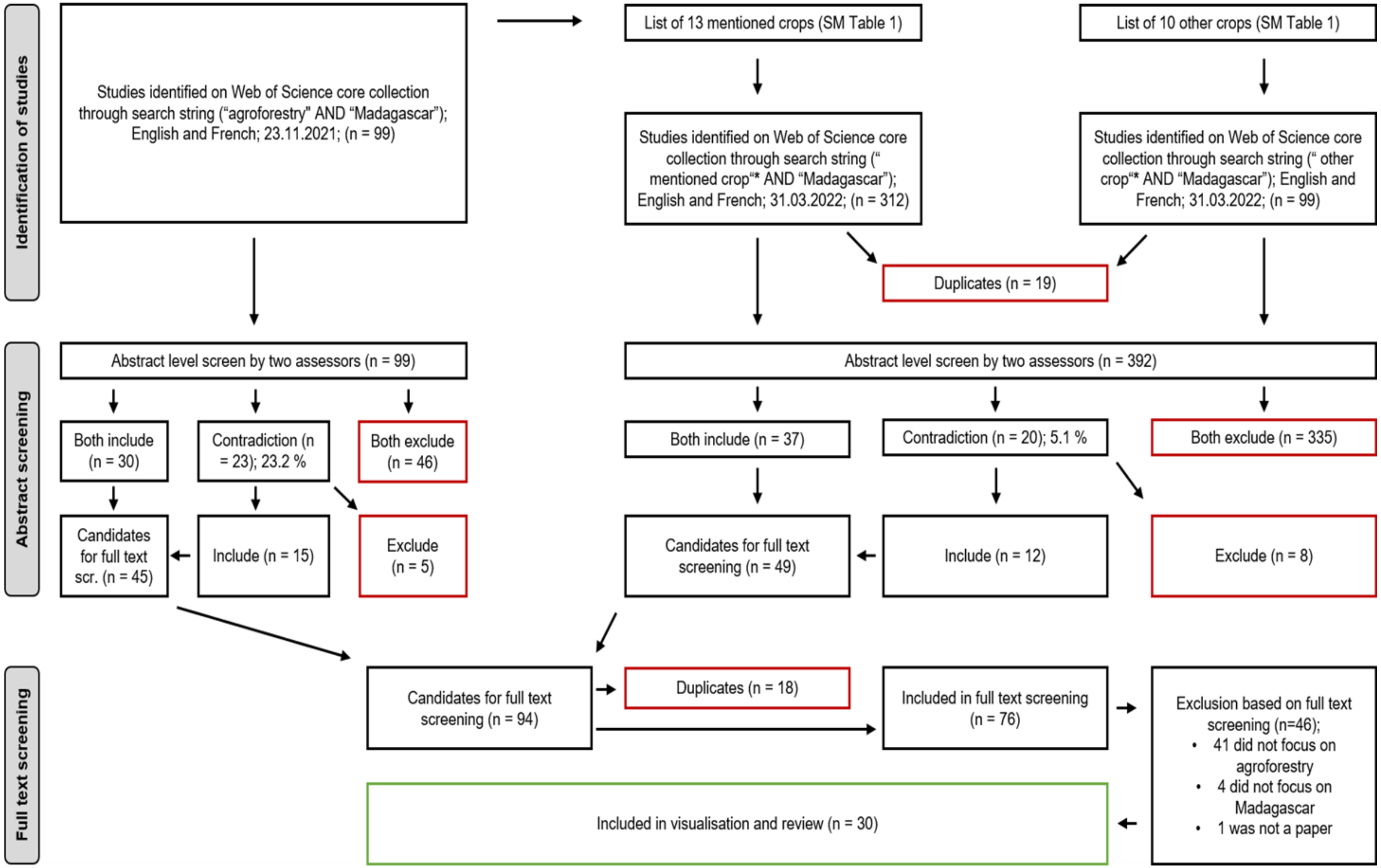

Figure S1 Flow diagram depicting identification and screening of studies with the respective number of studies that we excluded after scanning abstracts or full texts. Overall, we included 30 studies from an initial sample of 510 studies. The circumstance that two of the Web of Science searches resulted in 99 items each is coincidental.

Table S1. Three search strings applied in Web of Science to identify studies on agroforestry in Madagascar including query links to search results.

**Search**	**Search string**	**Query link**
Agroforestry & Madagascar	((agroforest OR agroforestry OR agroforesterie OR agroforet OR agroforestier) AND Madagascar	https://www.webofscience.com/wos/woscc/summary/5eb09c63-64d9-40ef-a995-fc963c966a56-14da5241/relevance/1
List of 14 crops from full text review	((((((((((((((((((((((((ALL=(coffee)) OR ALL=(banana)) OR ALL=(baobab)) OR ALL=(clove)) OR ALL=(vanilla)) OR ALL=(breadfruit)) OR ALL=(mango)) OR ALL=(jackfruit)) OR ALL=(cocoa)) OR ALL=(tsiperifery)) OR ALL=(lychee)) OR ALL=(avocado)) OR ALL=(coconut)) OR ALL=(litchi)) OR ALL=(cacao)) OR ALL=(café)) OR ALL=(banane)) OR ALL=(girofle)) OR ALL=(vanille)) OR ALL=(fruit à pain)) OR ALL=(mangue)) OR ALL=(jacquier)) OR ALL=(litchi)) OR ALL=(avocat)) OR ALL=(noix de coco)) OR ALL=(litchi)) AND ALL=(Madagascar)	https://www.webofscience.com/wos/woscc/summary/711914bd-48fe-458d-9961-2a6ca4359602-2dd378f2/relevance/1
List of 10 additional crops	(((((((((((((((ALL=(cashew)) OR ALL=(cinnamon)) OR ALL=(rubber)) OR ALL=(jatropha)) OR ALL=(pepper)) OR ALL=(ginger)) OR ALL=(khat)) OR ALL=(qat)) OR ALL=(citrus)) OR ALL=(ylang ylang)) OR ALL=(noix de cajou)) OR ALL=(cannelle)) OR ALL=(caoutchouc)) OR ALL=(poivre)) OR ALL=(gingembre)) OR ALL=(agrumes)) AND ALL=(Madagascar)	https://www.webofscience.com/wos/woscc/summary/095d2a95-44e7-4524-a4b5-29ce75a9c0a2-2dd3b79b/relevance/1

Note that special symbols like ê are ignored, so foret is the same as forêt
